# Emmer Wheat Eco-Geographic and Genomic Congruence Shapes Phenotypic Performance under Mediterranean Climate

**DOI:** 10.3390/plants11111460

**Published:** 2022-05-30

**Authors:** Aviya Fadida-Myers, Dana Fuerst, Aviv Tzuberi, Shailesh Yadav, Kamal Nashef, Rajib Roychowdhury, Carolina Paola Sansaloni, Sariel Hübner, Roi Ben-David

**Affiliations:** 1 Department of Vegetables and Field Crops, Institute of Plant Sciences, Agricultural Research Organization (ARO)—The Volcani Center, Rishon LeZion 7505101, Israel; aviya.fadida@mail.huji.ac.il (A.F.-M.); avivang@gmail.com (A.T.); shaileshagri9@gmail.com (S.Y.); kamal@volcani.agri.gov.il (K.N.); rajibroychowdhury86@gmail.com (R.R.); 2 The Robert H. Smith Faculty of Agriculture, Food and Environment, The Hebrew University of Jerusalem, Rehovot 7610001, Israel; 3 Galilee Research Institute (Migal), Tel-Hai Academic College, Upper Galilee 12210, Israel; danaf@migal.org.il (D.F.); sarielh@migal.org.il (S.H.); 4 Genetic Resource Program, International Maize and Wheat Improvement Center (CIMMYT), Carretera México-Veracruz Km. 45, El Batán, Texcoco 56237, Mexico; c.sansaloni@cgiar.org

**Keywords:** genetic variation, Mediterranean basin climate, emmer wheat, phenology, yield

## Abstract

Emmer wheat (*Triticum turgidum* ssp. *dicoccum*) is one of the world’s oldest domesticated crops, and it harbors a potentially rich reservoir of agronomic and nutritional quality trait variations. The growing global demand for plant-based health-food niche markets has promoted new commercial interest in ancient grains, including Emmer wheat. Although *T. dicoccum* can also perform well under harsh environments, its cultivation along the Mediterranean agro-ecosystems is sparse. Here, we analyze a unique tetraploid wheat collection (*n* = 121) representing a wide geographic range of Emmer accessions, using 9897 DArTseq markers and on-field phenotypic characterization to quantify the extent of diversity among populations and the interactions between eco-geographic, genetic, and phenotypic attributes. Population genomic inferences based on the DArTseq data indicated that the collection could be split into four distinguished clusters in accordance with their eco-geographic origin although significant phenotypic variation was observed within clusters. Superior early vegetative vigor, shorter plant height, and early phenology were observed among emmer wheat accessions from Ethiopia compared to accessions from northern regions. This adaptive advantage highlights the potential of emmer wheat as an exotic germplasm for wheat improvement through breeding. The direct integration of such germplasm into conventional or organic farming agro-systems under the Mediterranean basin climate is also discussed.

## 1. Introduction

Emmer wheat (*Triticum turgidum* ssp. *dicoccum*) is one of the oldest crops in the world and among the first plants to be domesticated in the Fertile Crescent region [1,2,3]. The origin of emmer wheat is the Levant near Tigris and the Euphrates rivers, modern day southeastern Turkey/northern Syria [4]. Ample archaeological evidence supports a diffusion process of emmer wheat starting from the Levant in Turkey, Greece, Bulgaria to southern Italy, southern France, and Spain along with the adoption of agricultural practices that have expanded substantially since 5500 BC [5]. Later, at approximately 3500 BC, emmer was introduced to northern Europe, including Germany, Poland, and the British Isles [6,7]. Approximately 500 years later, emmer wheat was introduced to Ethiopia via Egypt and Sudan [1,8,9].

The domesticated hulled emmer wheat is the direct progenitor of the tetraploid durum (*T. turgidum* ssp. *durum*) and both are allo-tetraploid species (2n = 4x = 28; genome BBAA) [10]. Historical evidence indicates that the cultivation of hulled ancient wheat species, including emmer, was gradually replaced with free threshing and high-yielding wheat varieties ending up today with modern elite varieties [11,12]. Hulled wheat such as einkorn (*T. monococcum* ssp. *monococcum* L.; genome AA), emmer, and spelt (*T. aestivum* spp. *spelta*; genome BBAADD) refer to wheat species that need further processing after harvest to release the kernel. Interestingly, despite the high yields obtained from modern wheat cultivars, emmer wheat is still sparsely grown under low input agro-systems across mountainous regions in several parts of Africa, West Asia, Southeast Europe, Ethiopia, and India [13,14]. The main reason hulled wheat is cultivated in marginal areas, except for conservation of local tradition, is their ability to maintain stable and satisfying yields also under harsh conditions. Moreover, ancient wheat grains are rich in dietary fiber and antioxidants, characterized by a low glycemic index, and considered hypoglycemic food [15]. It was also claimed that emmer grains can reduce the risk of chronic diseases such as heart disease, type 2 diabetes, obesity, and some forms of cancer [15]; thus, not surprisingly, ancient wheats such as emmer and spelt are gradually enjoying renewed interest among farmers, consumers, and bakers in Germany, Switzerland, Italy, and other countries in Europe [16].

In addition, emmer wheat germplasm can serve as a rich allelic reservoir for trait variation, including nutritional quality traits [17], biotic and abiotic stress resistance [18], phenological variation, and so forth. For example, recent work has highlighted emmer as a potential source for heat stress tolerance genes which could be introgressed into modern wheat without compromising important agronomic traits [19,20,21]. In another work, Ullah et al. [22] crossed emmer wheat with modern bread wheat, and they were able to increase grain yield by 7% under heat stress conditions.

Vernalization and photoperiod sensitivity both tune wheat flowering time and phenology, and consequently determine genotypic suitability for specific growing environments [23,24,25]. In the Mediterranean basin, these traits are particularly important due to unstable temperature and precipitation along the growing season [26,27,28,29]. Thus, the identification of suitable germplasm for the Mediterranean region can improve crop adaptability in rain-fed environments [16]. In the current study, we established a new set of emmer wheat derived from wide eco-geographical origins. High-throughput genotyping shed light on the population dynamics and their association with phenotypic performances under the Mediterranean climate basin. Our results will serve as the basis for future introgressing of new elite emmer cultivars adapted for the Mediterranean basin.

## 2. Results

### 2.1. Genetic Characterization of the Emmer Collection

**Figure 1:**
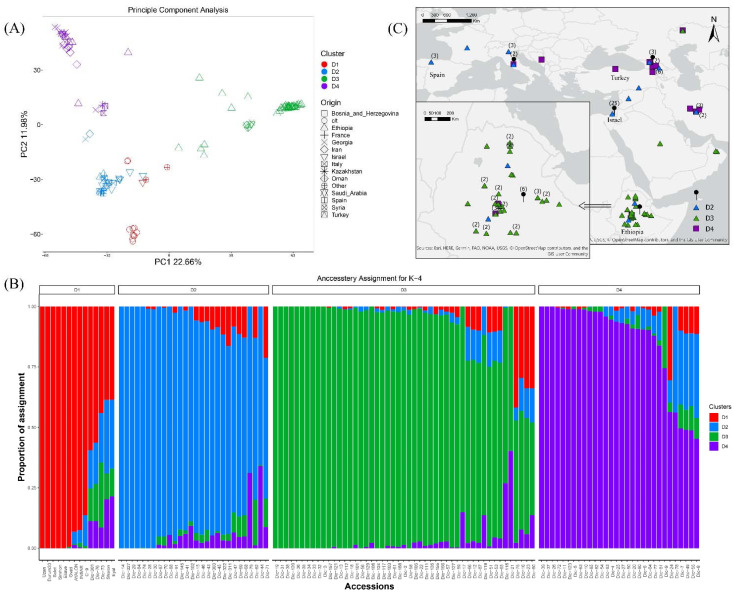
Genetic characterization of emmer wheat collection. (**A**) Principal component analysis (PCA) of individuals from various regions in Levant, Eurasia, and Ethiopia. The scatter plot consists of the two first PCs selected based on their highest eigenvalue. Country of origin is indicated with different shapes and their associated clustered (based on highest assignment in the sNMF analysis) is indicated with different colors. (**B**) A bar plot for the assignment of *T. dicoccum* and *T. durum* accessions to clusters as identified by the sNMF analysis for K = 4. In the bar plot, each bar corresponds to accession, and colors represent the assignment to each cluster. (**C**) The geographic distribution of the emmer wheat accessions. At the bottom left is a zoom-in view of accessions from Ethiopia. In the plot, different symbols and colors represent the assignment of accessions to a cluster, and the pinpoints denote accessions for which geographic coordinates were not available. Numbers in parentheses indicate different sampling sites in the same country of origin, cultivated accessions are not included.

All 135 genotypes comprising the emmer wheat collection were genotyped using the DArTseq procedure, yielding a total of 42,993 variants. After stringently filtering for low-quality variants and an excess of missing data, a total of 9897 SNPs genotyped across 121 accessions were kept for downstream analyses. Population stratification analyses based on sNMF (sparse nonnegative matrix factorization) followed by a cross validation test to identify the best K and PCA followed by a Tracy–Windom test to identify the best number of eigenvectors both indicated that the collection can be divided into four distinguishable clusters (Figure 1A,B, Figures S1 and S3). Generally, the population structure supported the expected *T. dicoccum*—*T. durum* split and, to a lower extent, the geographic distribution of accessions mainly between Ethiopian and Eurasian accessions. Interestingly, Levantine accessions were identified as an admixed population between Ethiopian, Mediterranean, and Eurasian types (Figure 1B), although signs of admixture were also observed among other clusters. As expected, the *T. durum* modern cultivars were clustered separately from rest of the collection (D1), yet two accessions from Ethiopia and one from Israel that were classified as emmer in the GenBank records were clustered together with durum cultivars indicating possible mislabeling of these accessions. All other emmer wheat accessions were divided into three distinguished clusters: cluster D2 (*n* = 28), mainly comprised of accessions from the Mediterranean region; cluster D3 (*n* = 49), mainly comprised of accessions originating from Ethiopia (83%), and the remaining accessions originated from Georgia, Israel, Kazakhstan, Oman, and Saudi Arabia; cluster D4 (*n* = 30) was comprised of accessions that originated from Bosnia and Herzegovina, Georgia, Iran, Italy, and Turkey (Figure 1C).

### 2.2. Phenotypic Characterization of the Emmer Collection

**Figure 2:**
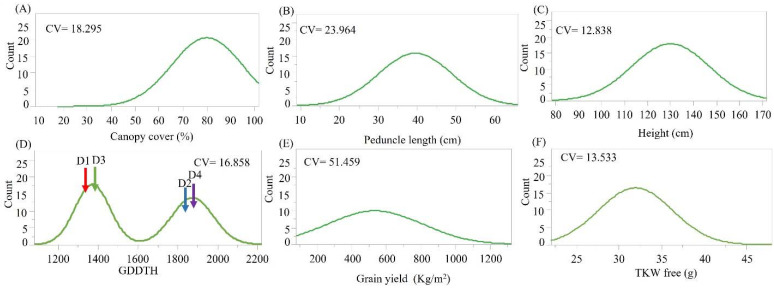
Distribution of phenotypic variables. (**A**) canopy cover (%); (**B**) peduncle length; (**C**) plant height; (**D**) GDD to heading; (**E**) grain yield; and (**F**) TKW. The coefficient of variation values is marked on each chart. Arrows represent cluster phenotypic mean.

**Figure 3:**
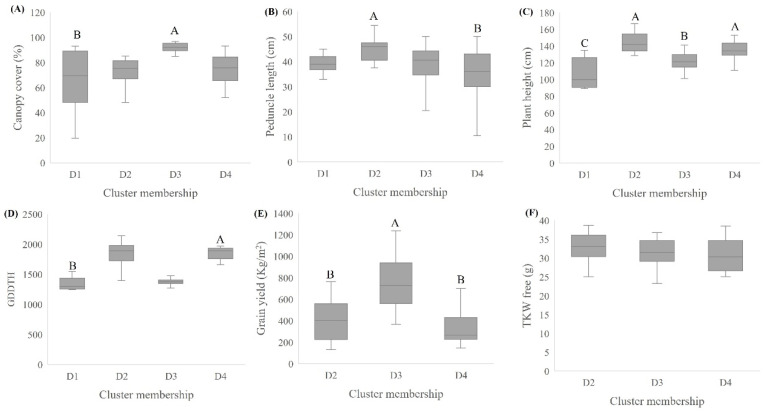
Box plot of agronomic traits by clusters: (**A**) canopy cover (%), (**B**) peduncle length, (**C**) plant height, (**D**) GDD to heading, (**E**) grain yield, and (**F**) TKW. Different letters, in the bars, indicate significant differences at *p* ≤ 0.05 following a mean comparison both Welch (**A**,**B**,**D**) tests were applied and Tukey–Kramer (**C**,**E**,**F**).

**Figure 4:**
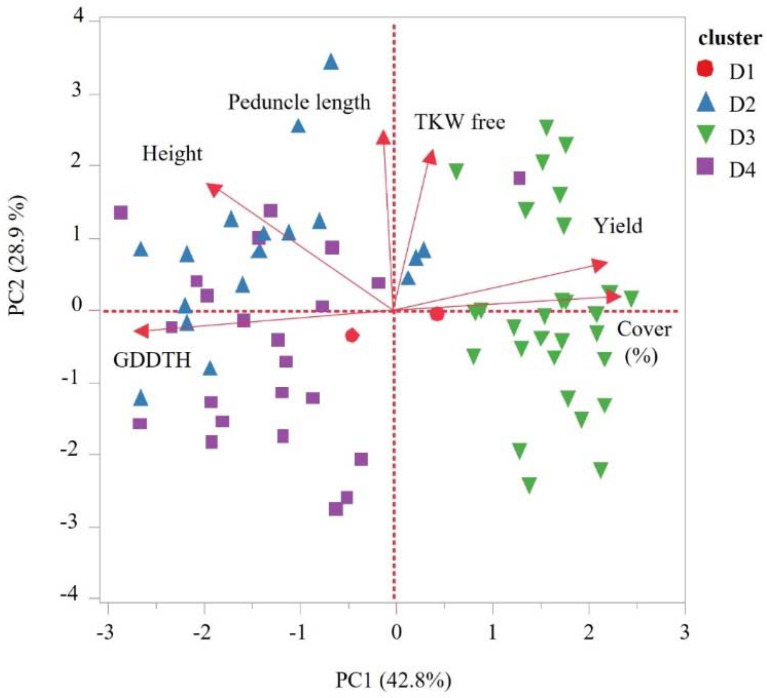
Scatter plot for the first two principal components obtained from PCA conducted using phenotypes of emmer wheat accessions evaluated in the field experiment. The PC factor loadings are indicated with arrows and the different shapes and colors represent the different genetic clusters. The phenotypic traits include canopy cover, peduncle length, plant height (Height), grain yield (GY), GDD to heading (GDDTH), and thousand-grain weight (TKW free). Shape and color represent membership of accessions to different genetic clusters D1(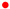), D2(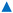), D3(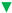), D4(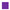).

The emmer wheat germplasm collection was grown in a common garden experiment. Early vigor and biomass productivity data expressed by canopy cover at early stages showed wide variation (Figure 2A). Most of the screened accessions showed canopy cover greater than 50% at 65 days after sowing (DAS), where accessions assigned to clusters D1 (including *T. durum* cultivars) and D3 (mainly Ethiopian *T. diccocum*) had the lowest (66.14%) and the highest (91.55%) canopy cover values, respectively (Figure 3A). Plant height and peduncle length had a unimodal normal distribution among accessions (Figure 2B,C), where accessions assigned to cluster D2 had a significantly longer peduncle compared with accessions assigned to cluster D4 (mean: D2 = 45.62 cm ± 1.51, D4 = 35.52 cm ± 2.09; Figure 3B; Table S2). In contrast, plant height was similar among D2 and D4 clusters, and both were significantly taller than D1 and D3 clusters (mean D2 = 144.676 cm ± 2.9, D4 = 136.04 cm ± 2.59, D1 = 106.25 cm ± 7.57, D3 = 121.65 cm ± 2.02; Figure 2C and Figure 3C; Table S4). Interestingly, flowering time corrected for GDD (growing degree days to heading -GDDTH) was characterized with bimodal distribution among accessions (Figure 2D); this phenology differentiation was in partial accordance with cluster membership. Generally, genotypes from D3 and D1 clusters were characterized by earlier flowering (D1 = 1341.43, D3 = 1365.31 GDD) than D2 and D4 clusters (D2 = 1834.42, D4 = 1840.57 GDD), although an overlap between distributions was observed (Figure 3D). Grain yield was also characterized with a bimodal distribution where clusters D3 (746.77 kg/m^2^) and D4 (351.39 kg/m^2^) had significantly higher and lower GY (grain yield) compared to all other clusters, respectively (F = 17.627, *p* < 0.0001; Figure 2E and Figure 3E). Lastly, grain weight (TKW) of free threshing grains was normally distributed with large differences between accessions (ranged between 23.26 g and 45.2 g), yet no significant differences were observed (F = 1.0975, *p* = 0.3564) between clusters (Figure 2F and Figure 3F, Table S2). To further evaluate the association between genetic clustering, geographic origin, and phenotypic characteristics, a principal component analysis was conducted based on the measured phenotypic values for each accession. The PCs explained 71.7% of the variance, enabling discrimination of accessions with partial accordance to the genetic clustering (Figure 4). The first PC explained 42.8% of the variance, and it was loaded positively by canopy cover (%), yield and TKW of free threshing grains and negatively by plant height, peduncle length, and GDDTH. The second PC explained 28.9% of the variance, and it was positively loaded mainly by peduncle length. The PCA depicted the negative correlation between canopy cover (%), GDDTH and plant height. Accessions that were assigned genetically to cluster D3 were concentrated at the right part of the PC plot in accordance with the early vigor growth (high canopy cover), early flowering (low GDDTH), and high GY and TKW observed for those accessions in the phenotypic characterization. In contrast, accessions that were assigned genetically to cluster D2 and D4 (mainly Eurasian accessions) were concentrated at the left side of the PCA plot, indicating a low early vigor, late phenology, and lower GY compared with other accessions (Figure 4).

### 2.3. The Interaction between Genetic Relatedness, Eco-Geographic Factors and Phenotypic Traits

**Figure 5:**
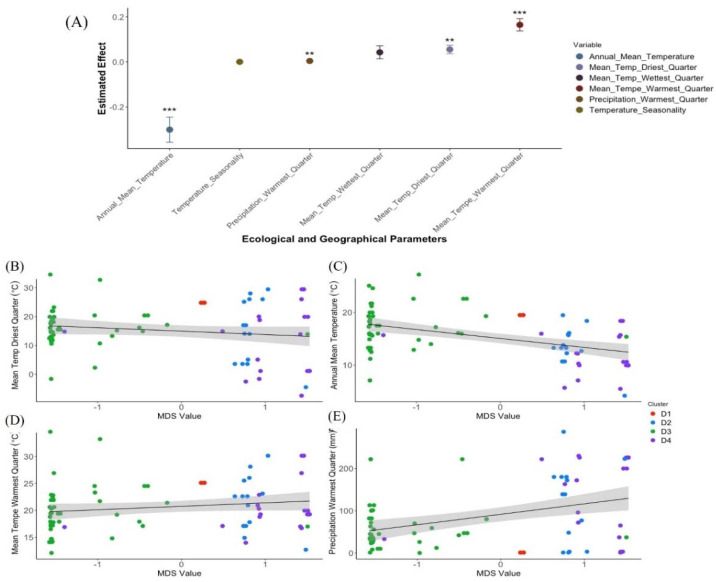
Linear regression models for the effect of ecogeographical parameters on genetic relatedness. (**A**) The estimated effect and the residuals for each of the six parameters included in the linear model,). *** represents the significance level *p* < 0.0001; ** represents the same *p* < 0.001. (**B**–**E**) Scatter plots between the genetic relatedness calculated with MDS and temperature range and precipitation parameters. Each cluster is denoted with a different color.

**Figure 6:**
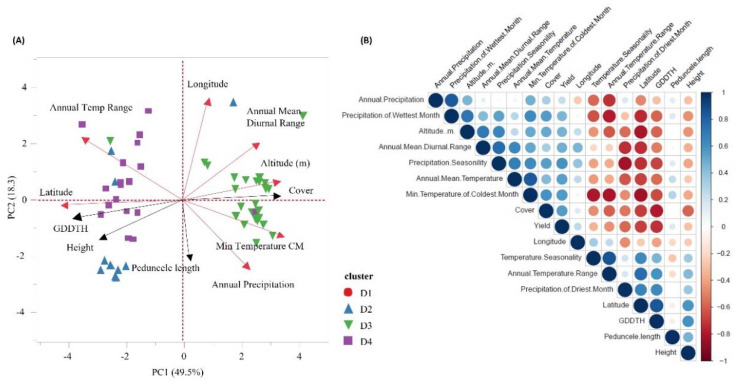
(**A**) Principal component analysis (PCA) of eco-geographic and phenotypic data. PCA of phenotypic (black arrow: cover, height, GDDTH) and eco-geographic traits (red arrow: annual temperature range, latitude, annual precipitation, minimum temperature of the coldest month (CM), altitude (m), annual mean diurnal range, and longitude). Shape and color represent accessions membership to different genetic clusters D1(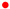), D2(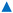), D3(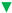), D4(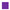). (**B**) Pearson correlation matrix of all tested traits. Bold values represent statistical significance (*p* ≤ 0.05). Colors indicate the level of correlation (r) from positive correlation (blue) to negative (red). Circle size indicates the level of significance, and the bigger and darker color dot represents significant correlations at *p* < 0.05.

Among the 121 accessions included in the study, geographic coordinates were available for 79 genotypes, thus regression analyses between eco-geographic factors and genetic similarity included only those accessions. A correlation test was performed among all 22 eco-geographic parameters obtained from the WorldClim database. Highly correlated variables were excluded to avoid bias due to autocorrelation in the linear model. Thus, seventeen parameters with a correlation coefficient lower than 0.8 were included in an iterative multi-regression modeling procedure (stepAIC) to identify a linear model that best fit the data (Table S3). Following this procedure, the best model (AIC = −29.7545, Table S3) was chosen, and it included the following parameters: annual mean temperature (BIO1), temperature seasonality (BIO4), mean temperature during the wettest quarter (BIO8), mean temperature during the driest quarter (BIO9), mean temperature in the warmest quarter (BIO10), and precipitation (BIO12). A multi-regression model was conducted to estimate the effect of selected parameters on the level of relatedness among accessions (Figure 5, Table S4). Overall, the eco-geographic parameters that were included in the model explained more than 60% of the variation in genetic relatedness (r^2^ = 0.6108, *p*  =  8.052 × 10^−14^), where mean temperature (*p* = 7.21 × 10^−7^) and precipitation (*p* = 5.65 × 10^−3^) had the strongest effect on the genetic composition (Figure 5, Table S3). Following the multi-regression model, a linear regression analysis was conducted for each eco-geographic parameter and genetic relatedness. In all models, a clear separation was observed between cluster D3 and all other clusters (Figure 5B–E). To further validate, the observation that temperature and precipitation are the main eco-geographic factors that differentiate between clusters, a PCA was conducted for all available phenotypic traits and eco-geographic parameters. The discrimination between cluster D3 and the remaining clusters was supported genetically, phenotypically, and eco-geographically, indicating that the Ethiopian accessions (cluster D3) represent a diverged clade of emmer wheat (Figure 6A). The first PC explained 49.5% of the variance, and it was positively loaded with peduncle length, annual precipitation, minimum temperature, canopy cover, altitude, annual mean diurnal range, and longitude, and negatively loaded with peduncle length, plant height, GDDTH, latitude, and annual temperature range. The separation between cluster D3 and the remaining clusters is explained mainly by the factors associated with PC1 (Figure 6A). This multi-correlation analysis highlights the association between eco-geographic variables and phenotypic traits (Figure 6B). Latitude was significantly and positively correlated with GDDTH (r = 0.84) and significantly and negatively correlated with plant cover (r = −0.78) (Figure 6B, Table S5). GDDTH was significantly and negatively correlated with temperature measurements [annual mean diurnal range (r = −0.491), annual mean temperature (r = −0.58), and minimum temperature in the coldest month (r = −0.67)] (Figure 6B, Table S5).

## 3. Discussion

Wheat is one of the founder grain-crops of the Near East, and it is being cultivated today across a wide range of environments worldwide. Wheat heading the list for the most widely grown crops; thus, a lot of attention and breeding efforts are invested in this crop. Emmer wheat, an ancient grain crop, is attracting more attention in recent years due to its high nutritional value and potentially beneficial diversity which could be used to enrich the allelic repertoire for wheat breeding programs [16,22]. In this study, we combined phenotypic characterization under Mediterranean field conditions and DArTseq-based genetic profiling to investigate the adaptive potential of emmer accessions under Mediterranean conditions.

The diffusion of emmer wheat to Europe is intertwined with the spread of agricultural practices [5]. Vavilov [30] and Gokgol [31] have previously divided emmer gene pool into four sections. Interestingly, our genomic analysis also generated four clusters (D1-4), with some overlap with Vavilov’s systematic categorization. The D3 cluster resembles the Abyssinian emmer (Subsp. Abyssinicum Vav.), which consists mainly of spring type, early flowering (Figure 2D and Figure 3D), and short stature emmer accessions (Figure 2C). This cluster is comprised mainly of accessions originating from Ethiopia (80%), similar to Abyssinian emmer [18]. The low genomic admixture observed in the D3 cluster (Figure 1B) implies that these accessions went through a genetic bottleneck during their introduction to Ethiopia, presumably from the Mediterranean basin 5000 years ago (Figure 1C). Accessions that correspond to the D4 cluster share similarities with Vavilov’s definition of European emmer [30], which is characterized by late flowering and high plant stature (Figure 3C,D). Gene-flow between geographic regions may be the main cause for the high genetic admixture observed between the Eastern (subs. Asiaticum Vav.) and European emmer (Subsp. dicoccon) corresponding cluster D2 (Figure 1). Nevertheless, cluster D2 includes mainly germplasm of eastern emmer type which fits Zaharieva et al. [19] classifications. Accessions in the D2 cluster are characterized by intermediate phenology and a wide range of plant height (intermediate to high) (Figure 2C and Figure 3C,D). The fourth Vavilov [30] section, Moroccan emmer (Subsp. Maroccanum Flaksb.), is not represented in our collection. Further work should emphasize exploring this interesting emmer group which potentially harbors beneficial alleles that can enhance adaptation to Mediterranean environments, including very early flowering, high tillering, and short stature [19].

Our analyses, including the linear model, suggest an association between genetic, phenotypic, and geographic distribution (Tables S3 and S4), which is in contrast with Wang et al. [32] and Teklu et al. [33] who did not find any significant association between these factors among geographically diverse emmer collections (91 and 73 accessions, respectively). In addition, our multivariate analysis revealed significant correlations between temperature and precipitation variables with yield and development traits, including grain yield, canopy cover, and plant height (Figure 6, Table S5). The pivotal role of earliness is adaptation to Mediterranean growing conditions [16], which are characterized by a short winter season that is terminated by dry and hot sprints. Day length insensitivity was identified as a key adaptive trait, and it has a clear advantage in rain-fed Mediterranean fields. Thus, we have prioritized accessions originating from low latitudes, assuming these genotypes will reflect negative selection to day length sensitivity. Indeed, most of the accessions comprising cluster D3 originated from low latitude and early flowering, similar to the Mediterranean modern durum cultivars of cluster D1 that are adapted to those conditions (Figure 2D and Figure 3D). Therefore, D3 emmer genotypes exhibit phenotypic superiority under Mediterranean environments mainly because of early phenology, and they are consequently excelling in GY (Figure 3E, Figure 4E and Figure 6A). We can also assume D3 GY superiority is derived from a higher grain number per plant as TKW was not significantly different between the clusters (Figure 3F). In addition, accessions from cluster D3 had shorter stature, similar to the semi-dwarf modern durum cultivars (Figure 3C), minimizing their susceptibility to lodging and its negative impact on grain yield. Longin et al. [34] showed large genetic variation in plant height and GY, with no negative correlation between them. In our study, no significant correlation was detected between these traits, although plant height was negatively correlated with canopy cover and positively correlated with GDDTH (Figure 6B, Table S5). Moreover, GDDTH was negatively correlated with yield (Figure 4) in the Mediterranean environment, and the phenology and early vigor had more influence on yield than plant height.

Early vigor is an important trait under Mediterranean climate, providing several benefits for short winter seasons. Early high canopy cover observed among accessions comprising the cluster D3 (Figure 3A) can reduce water evaporation from the soil [35,36], enhance competitiveness with weeds [37,38,39], and increase radiation-use efficiency (RUE) [40,41]. This is also reflected by the significant correlation between canopy cover and yield (r = 0.45; Figure 4).

To summarize, this study evidently shows the adaptive potential of emmer germplasm originating from Ethiopia (D3 cluster) to grow in a Mediterranean climate. However, to further approve this potential, a profound and a broader agronomic examination (e.g., testing a core set of emmer accessions under a multi-year, multi-environmental setup) is needed. Such work should target not only productivity but also grain quality because emmer will be adapted as a crop by local farmers, millers, bakers, and customers only if they are convinced in both of these aspects. In addition, emmer wheat as well as other hulled wheat types require the further post-harvest grain processing step of dehulling. As such, it requires investment in specialized dehulling machinery in order to insure a sequential food chain. Finally, economic benefits and grain quality attributes in comparison to the common crops bread and durum wheat will be the dominant drivers of emmer adaptation in a Mediterranean environment.

## 4. Materials and Methods

### 4.1. Plant Materials

A collection of 124 accessions of emmer wheat representing 13 different geographical regions (Ethiopia, Iran, Oman, Saudi Arabia, Syria, Israel, Kazakhstan, Bosnia Herzegovina, France, Georgia, Italy, Spain, and Turkey) was established from seeds provided by the National Small Grains Collection (NSGC), USDA. Eleven Israeli modern durum wheat cultivars, (Inbar, Sharon, Solet, Eyal, Simhon, Eliave, Uzan, Gvati, Ayalon, C-9, and Durum33) were included in the collection as a control (Table S1). The germplasm used in this study complies with institutional, national, and international guidelines and legislation of plant material.

### 4.2. Eco-Geographic Profile of the Germplasm Collection

Geographic coordinates were available for most accessions (79 accessions); thus, data mining was performed following Ronen et al. [30], using this sub-set. Based on geographic coordinates (extracting from “wheat gateway [31]” based on PI line number), climatic data were retrieved from the WorldClim database [32] at 1 km spatial resolution, and it included 20 parameters: annual mean temperature (BIO1), annual mean diurnal range (BIO2), isothermality (BIO3), temperature seasonality (BIO4), max temperature of warmest month (BIO5), min temperature of coldest month (BIO6), annual temperature range (BIO7), mean temperature of wettest quarter (BIO8), mean temperature of driest quarter (BIO9), mean temperature of warmest quarter (BIO10), mean temperature of coldest quarter (BIO11), annual precipitation (BIO12), precipitation of wettest month (BIO13), precipitation of driest month (BIO14), precipitation seasonality (BIO15), precipitation of wettest quarter (BIO16), precipitation of driest quarter (BIO17), precipitation of warmest quarter (BIO18), precipitation of coldest quarter (BIO19), and altitude (m) [32].

### 4.3. Genotyping and Population Structure Analysis

DArTseq genotyping of the emmer collection was performed at the International Maize and Wheat Improvement Center (CIMMYT; see Sansaloni et al. [33] for details) using a total of 42,993 SNP markers. The called variant dataset was converted to a vcf format using plink2 [34], and it was filtered for a minimum minor allele frequency of 5% and a maximum of 40% missing calls after excluding individuals with an excess of missing data (>70%) using vcftools V.0.1.15 [35]. Population stratification among emmer accessions was tested using a principal component analysis (PCA) followed by a Tracy–Widom (TW) test (Figure S1) to choose the best number of eigenvectors that represent the data that corresponds to the number of clusters. In addition, a sNMF (sparse nonnegative matrix factorization) analysis was conducted with several clusters (K) ranging from 1 to 10 followed by a cross-validation test to choose the best number of clusters (K) representing the data (Figure 1B and Figure S3). Both population structure analyses were conducted with the R package LEA V-2.0 [36].

### 4.4. Field Assay and Phenotypic Characterization

A common garden experiment was conducted at Avnei Eitan experimental station (32°82′ N, 35°76′ E; 385 MASL), Eastern Israel, during the 2017–2018 growing season. This site is characterized by a Mediterranean climate with an average annual rainfall of 460 mm (Figure S2) and clay-heavy soil. The experiment included 87 plots (one for each genotype), the size of each plot was 2 m^2^, with four rows per plot (25 cm space between rows). Sowing was performed manually on 27 November 2017, using a Chapin Garden Push seeder (8701B, Australia). The experimental field was treated with fungicides (Folicur^®^ EC 250@750 mL/ha) and pesticides (Talstar^®^ a.i. Bifenthrin@ 500 mL/ha) to prevent infestation of fungal pathogens. In addition, an insect pesticide was implemented 67 days after emergence. Manual weeding was performed in periodic intervals of three weeks during the crop growth. The recommended dose of nitrogen was applied (urea 300 kg/ha) in three equal doses: before sowing, at tillering stage (2 months after sowing), and before heading (3 months after sowing). Plots were characterized phenotypically for the following traits: canopy leaf cover (%) was determined 65 days after sowing by a cell-phone based automated digital image analysis application (Easy Leaf Area37); days to heading was recorded when 80% of spikes in a given plot were visible, and growing degree days (GDDTH) for heading was calculated. Plant height (Height) was measured from three random main stems per plot at maturity (measuring from the soil surface to spike tip excluding awns). The peduncle length was measured from the last node to the base of the spike. The grain yield (GY) was calculated per plot after harvesting manually and calculating yield. The thousand kernel weight (TKW) was measured when plants were at 12% moisture content for both hulled and free-threshing grains. Bird damage was observed for seven accessions; thus, these accessions were excluded in the calculation of GY and TKW.

### 4.5. Statistical Analysis

Statistical analysis of phenotype data was performed using JMP^®^ ver. 15.0 pro statistical package (SAS Institute, Cary, NC, USA), and it included descriptive statistics for the full dataset and analysis of variance (ANOVA). To test the effect of eco-geographic factors on the level of genetic similarity among accessions, a multivariate linear model analysis was applied. To obtain a unidimensional score that represented the genetic composition of each individual, a multidimensional scaling (MDS) was conducted, with the “cmdscale” function in R on the pair-wise relatedness matrix calculated from the identity by state, using the SNPrelate package [36]. This procedure allowed us to obtain a single score for each accession which represented its similarity to other accessions (close scores indicate higher relatedness), which was used as a response variable in the linear model. To avoid bias in the linear model due to autocorrelation between environmental variables, a Pearson correlation test was performed between all pairs of parameters. When high correlation was observed (r > 0.8), only one of the parameters (lowest rate of missing data) was kept in the linear model. All remaining eco-geographic parameters were used to determine the best parsimonious model based on a stepAIC algorithm as implemented in the MASS V-7.3-53.1 package in R [38]. The stepAIC algorithm iteratively tests all possible combinations of parameters, and it reports an Akaike information criterion (AIC) score for each model, thus allowing the selection of the model that best fits the data.

## 5. Conclusions

The Mediterranean environment is characterized by a short, warm, and unstable precipitation, thus cultivated crops should be well adapted to this short life cycle. The strong negative correlation between early vigor and GY with GDDTH detected in the current study, supports this argument. The geographic origin of emmer accessions had a strong impact on the genetic profile and on plant performance, and it was probably strongly shaped by evolvement under traditional agriculture for several millennia. Emmer wheat accessions originating from Ethiopia performed superiorly under Mediterranean climates, and it should be prioritized in future breeding efforts in this target region.

## Data Availability

R scripts will be available through github https://github.com/hubner-lab (accessed on 1 May 2022).

## Supplementary Materials

The following supporting information can be downloaded at: https://www.mdpi.com/article/10.3390/plants11111460/s1, Figure S1. Tracy-Widom (TW) test the best number of clusters that represent the collection represented by the brake point of the graph (4). Figure S2. Meteorological data profile of 2017-2018 growing season in Avni Eitan site (32.82, 35.76; 385 m asl). Arrows. represent irrigation, a Total of 461mm of rain and 40mm of supplementary irrigation. Table S1. list of 121 collection line. code list for this research (Dic code), serial number at gene bank (serial), common name (plant ID/ name), country of origin (country), genetic cluster classified (cluster) and fields tested status (planted). Table S2. Phenotypic traits mean, standard error, and range of values of the four genetic clusters (canopy cover, peduncle length, height, GDDTH, grain yield and TKW free grain). Table S3. Akaike information criterion (AIC) analysis on mixed linear model containing all 13 parameters with correlation < 0.8. summer of square (sum of Sq), Residual sum of squares (RSS), Akaike information criterion (AIC) model value. Table S4. Results of the most fitted mixed linear model, the effect of geographical and ecological on the genetic variation. Estimate effect of each ecogeographic parameter (Estimate), standard error (Std. error), t value of each parameter in the mixed linear model and probability (P > |t|). Table S5. Ecogeographic (latitude, longitude, altitude, Annual Mean Diurnal Range, Annual Mean Temperature, Min Temperature Cold month, Annual Temperature Range and Annual Precipitation) and phenotypic (canopy cover, peduncle length, height, GDDTH, and grain yield) data correlation. Red marker significant under *p* < 0.05.

## References

[1] Damania A.B., Hakim S., Moualla M.Y. (1992). Evaluation of variation in *Triticum dicoccum* for wheat improvement in stress environments. Hereditas.

[2] Nesbitt M. (1996). From staple crop to extinction? The archaeology and history of hulled wheat. Hulled Wheat: Promoting the Conservation and Use of Underutilized and Neglected Crops.

[3] Hammer K., Filatenko A.A., Alkhanjari S., Al-Maskri A., Buerkert A. (2004). Emmer (*Triticum dicoccon Schrank*) in Oman. Genet. Resour. Crop Evol..

[4] Lev-Yadun S., Gopher A., Abbo S. (2000). Archaeology. The cradle of agriculture. Science.

[5] Kipfer B.A. (2000). Encyclopedic Dictionary of Archaeology.

[6] Ammerman A.J., Cavalli-Sforza L.L. (1984). The Neolithic Transition and the Genetics of Populations in Europe.

[7] Barker G. (1985). Prehistoric Farming in Europe.

[8] Helbaek H.H., Mellaart J. (1970). The plant husbandry of Haçilar. Excavations at Haçilar.

[9] Feldman M., Simmonds N.W. (1979). Wheats (*Triticum* spp.). Evolution of Crop Lants.

[10] Feldman M. (2001). The Origin of Cultivated Wheat.

[11] Feldman M., Kislev M.E. (2007). Domestication of emmer wheat and evolution of free-threshing tetraploid wheat. Isr. J. Plant Sci..

[12] Giuliani A., Karagöz A., Zencirci N. (2009). Emmer (*Triticum dicoccon*) Production and Market Potential in Marginal Mountainous Areas of Turkey. Mt. Res. Dev..

[13] Troccoli A., Codianni P. (2005). Appropriate seeding rate for einkorn, emmer, and spelt grown under rainfed condition in southern Italy. Eur. J. Agron..

[14] Oliveira H.R., Jones H., Leigh F., Lister D.L., Jones M.K., Peña-Chocarro L. (2011). Phylogeography of einkorn landraces in the Mediterranean basin and Central Europe: Population structure and cultivation history. Archaeol. Anthropol. Sci..

[15] Dhanavath S., Prasada Rao U.J.S. (2017). Nutritional and Nutraceutical Properties of *Triticum dicoccum* Wheat and Its Health Benefits: An Overview. J. Food Sci..

[16] Curzon A.Y., Kottakota C., Nashef K., Abbo S., Bonfil D.J., Reifen R., Bar-El S., Rabinovich O., Avneri A., Ben-David R. (2021). Assessing adaptive requirements and breeding potential of spelt under Mediterranean environment. Sci. Rep..

[17] Čurna V., Lacko-Bartoosova M. (2017). Chemical Composition and Nutritional Value of Emmer Wheat (*Triticum dicoccon Schrank*): A Review. J. Cent. Eur. Agric..

[18] Peng J., Sun D., Peng Y., Nevo E. (2013). Gene discovery in *Triticum dicoccoides*, the direct progenitor of cultivated wheats. Cereal Res. Commun..

[19] Zaharieva M., Ayana N.G., Hakimi A.A., Misra S.C., Monneveux P. (2010). Cultivated emmer wheat (*Triticum dicoccon Schrank*), an old crop with promising future: A review. Genet. Resour. Crop Evol..

[20] Nevo E. (2014). Evolution of wild emmer wheat and crop improvement. J. Syst. Evol..

[21] Trethowan R.M. (2014). Delivering drought tolerance to those who need it: From genetic resource to cultivar. Crop Pasture Sci..

[22] Ullah S., Bramley H., Mahmood T., Trethowan R. (2021). Implications of emmer (*Triticum dicoccon Schrank*) introgression on bread wheat response to heat stress. Plant Sci..

[23] Zheng B., Biddulph B., Li D., Kuchel H., Chapman S. (2013). Quantification of the effects of VRN1 and Ppd-D1 to predict spring wheat (*Triticum aestivum*) heading time across diverse environments. J. Exp. Bot..

[24] Yan L., Helguera M., Kato K., Fukuyama S., Sherman J., Dubcovsky J. (2004). Allelic variation at the VRN-1 promoter region in polyploid wheat. Theor. Appl. Genet..

[25] Beales J., Turner A., Griffiths S., Snape J.W., Laurie D.A. (2007). A Pseudo-Response Regulator is misexpressed in the photoperiod insensitive Ppd-D1a mutant of wheat (*Triticum aestivum* L.). Theor. AppL. Genet..

[26] Hyles J., Bloomfield M.T., Hunt J.R., Trethowan R.M., Trevaskis B. (2020). Phenology and related traits for wheat adaptation. Heredity.

[27] Bonfil D.J., Mufradi I., Klitman S., Asido S. (1999). Wheat Grain Yield and Soil Profile Water Distribution in a No-Till Arid Environment. Agron. J..

[28] Bonfil D.J., Abbo S., Svoray T. (2015). Sowing date and wheat quality as determined by gluten index. Crop Sci..

[29] Miller O., Helman D., Svoray T., Morin E., Bonfil D.J. (2019). Explicit wheat production model adjusted for semi-arid environments. Field Crops Res..

[30] Vavilov N.I. (1964). Plant immunity to infectious diseases. Izbrannye Trudy (Selected Works).

[31] Gokgol M. (1955). Bugdaylarin Tansif Anahtari. Ziraat Vekaleti Yayin.

[32] Wang X., Li W., Zheng Z. (2007). Principal component and cluster analysis of agronomic characters in *Triticum dicoccum Schrank*. J. Sichuan Agric. Univ..

[33] Teklu Y., Hammer K., Röder M.S. (2007). Simple sequence repeats marker polymorphism in emmer wheat (*Triticum dicoccon Schrank*): Analysis of genetic diversity and differentiation. Genet. Resour. Crop Evol..

[34] Longin C.F.H., Ziegler J., Schweiggert R., Koehler P., Carle R., Würschum T. (2016). Comparative study of hulled (einkorn, emmer, and spelt) and naked wheats (durum and bread wheat): Agronomic performance and quality traits. Crop Sci..

[35] Rebetzke G.J., Richards R.A. (1999). Genetic improvement of early vigour in wheat. Aust. J. Agric. Res..

[36] Lemerle D., Gill G.S., Murphy C.E., Walker S.R., Cousens R.D., Mokhtari S., Peltzer S.J., Coleman R., Luckett D.J. (2001). Genetic improvement and agronomy for enhanced wheat competitiveness with weeds. Aust. J. Agric. Res..

[37] Mason H.E., Spaner D. (2006). Competitive ability of wheat in conventional and organic management systems: A review of the literature. Can. J. Plant Sci..

[38] Reiss A., Fomsgaard I.S., Mathiassen S.K., Kudsk P. (2018). Weed suppressive traits of winter cereals: Allelopathy and competition. Biochem. Syst. Ecol..

[39] Aharon S., Fadida-Myers A., Nashef K., Ben-David R., Lati R.N., Peleg Z. (2021). Genetic improvement of wheat early vigor promote weed-competitiveness under Mediterranean climate. Plant Sci..

[40] Richards R.A. (1991). Crop improvement for temperate Australia: Future opportunities. Field Crops Res..

[41] Lemerle D., Verbeek B., Cousens R.D., Coombes N.E. (1996). The potential for selecting wheat varieties strongly competitive against weeds. Weed Res..

