# Supplementary figures and images for: Emmer Wheat Eco-Geographic and Genomic Congruence Shapes Phenotypic Performance under Mediterranean Climate
Source: Plants (Basel). 2022 May 30;11(11):1460. doi: 10.3390/plants11111460 (PMC9183160; doi:10.3390/plants11111460)

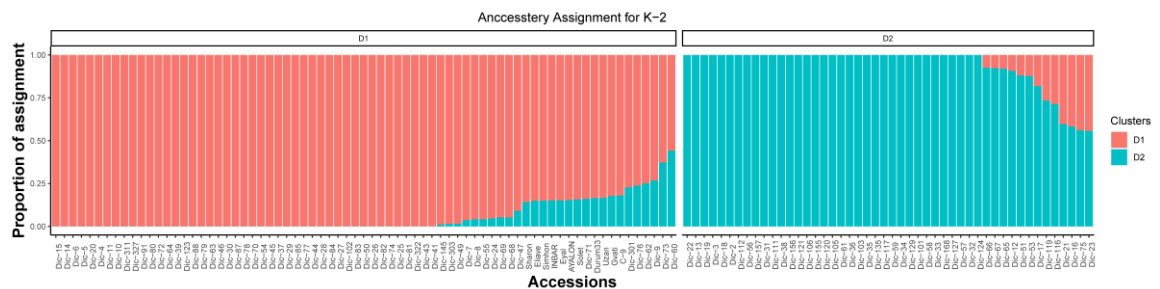

(A)

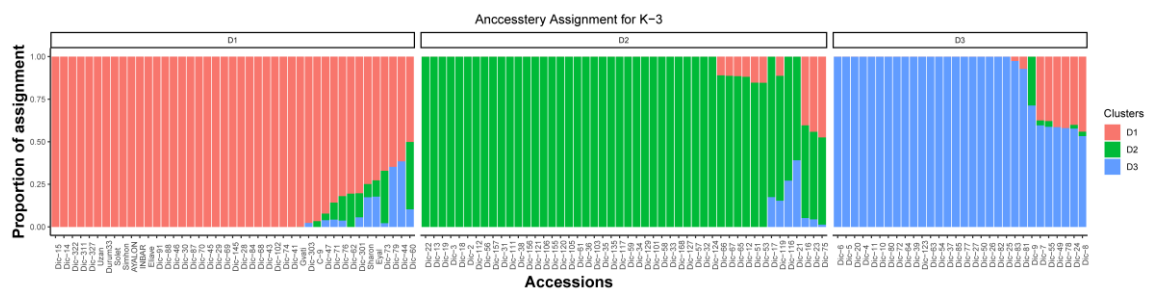

(B)

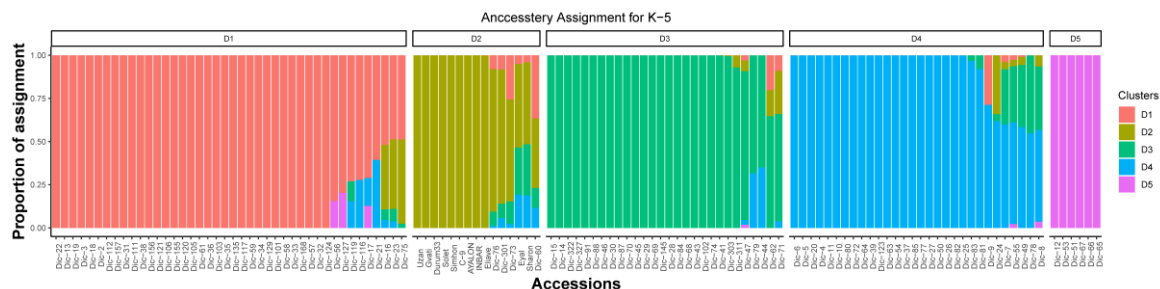

(C)

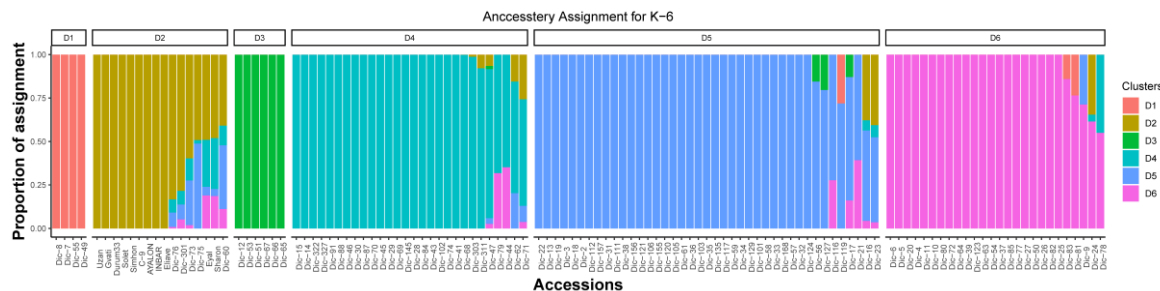

(D)

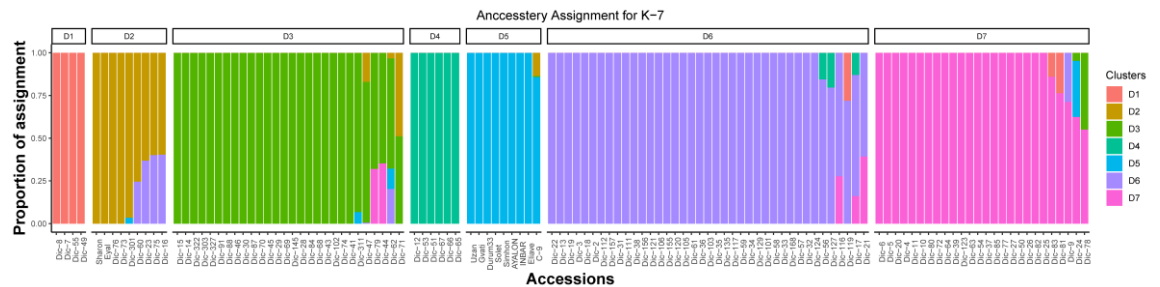

(E)

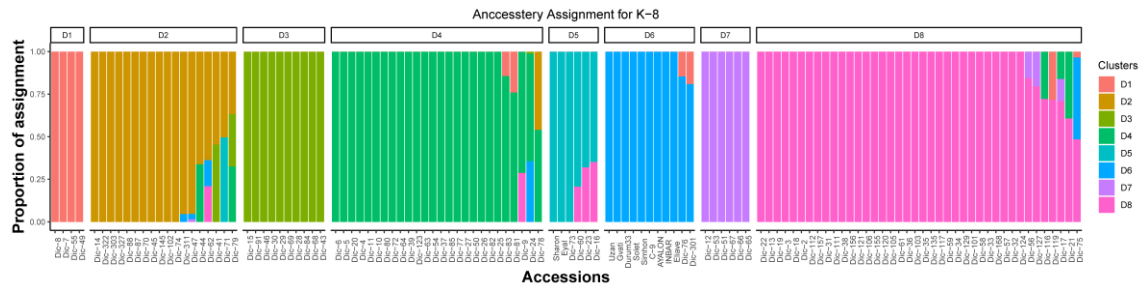

(F)

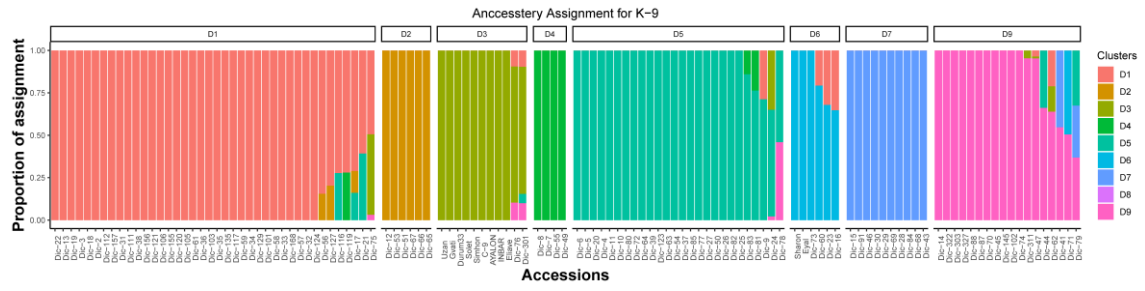

(G)

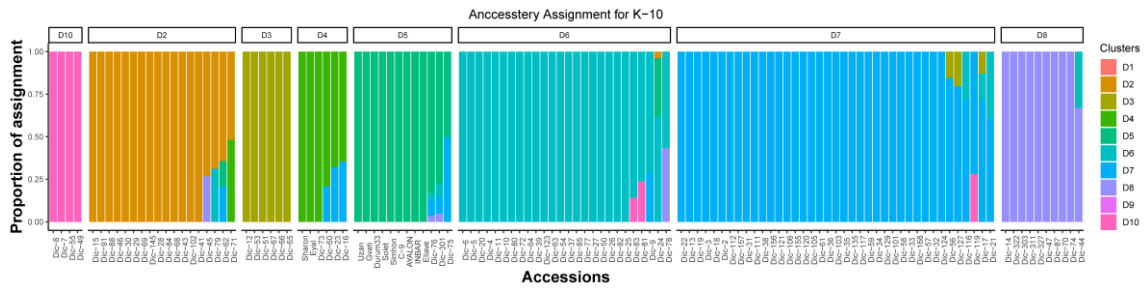

(H)

Supplement: Supplementary file 1 [file plants-11-01460-s001.zip › Supplementary Figures/Figure S3.pdf]

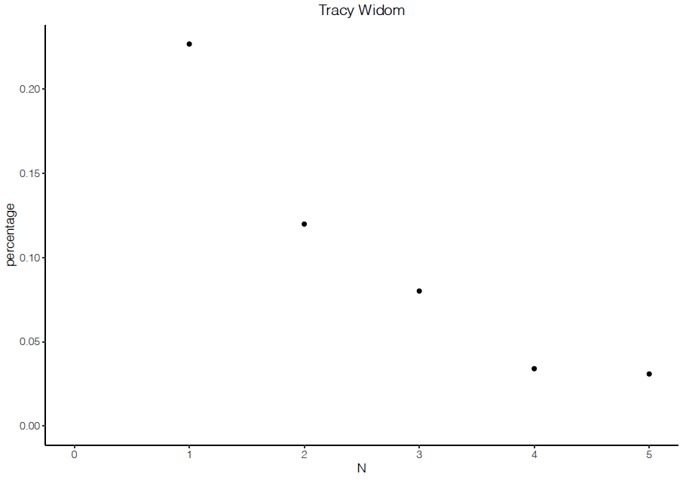

Supplement: Supplementary file 1 [file plants-11-01460-s001.zip › Supplementary Figures/Figure.S1.jpg]

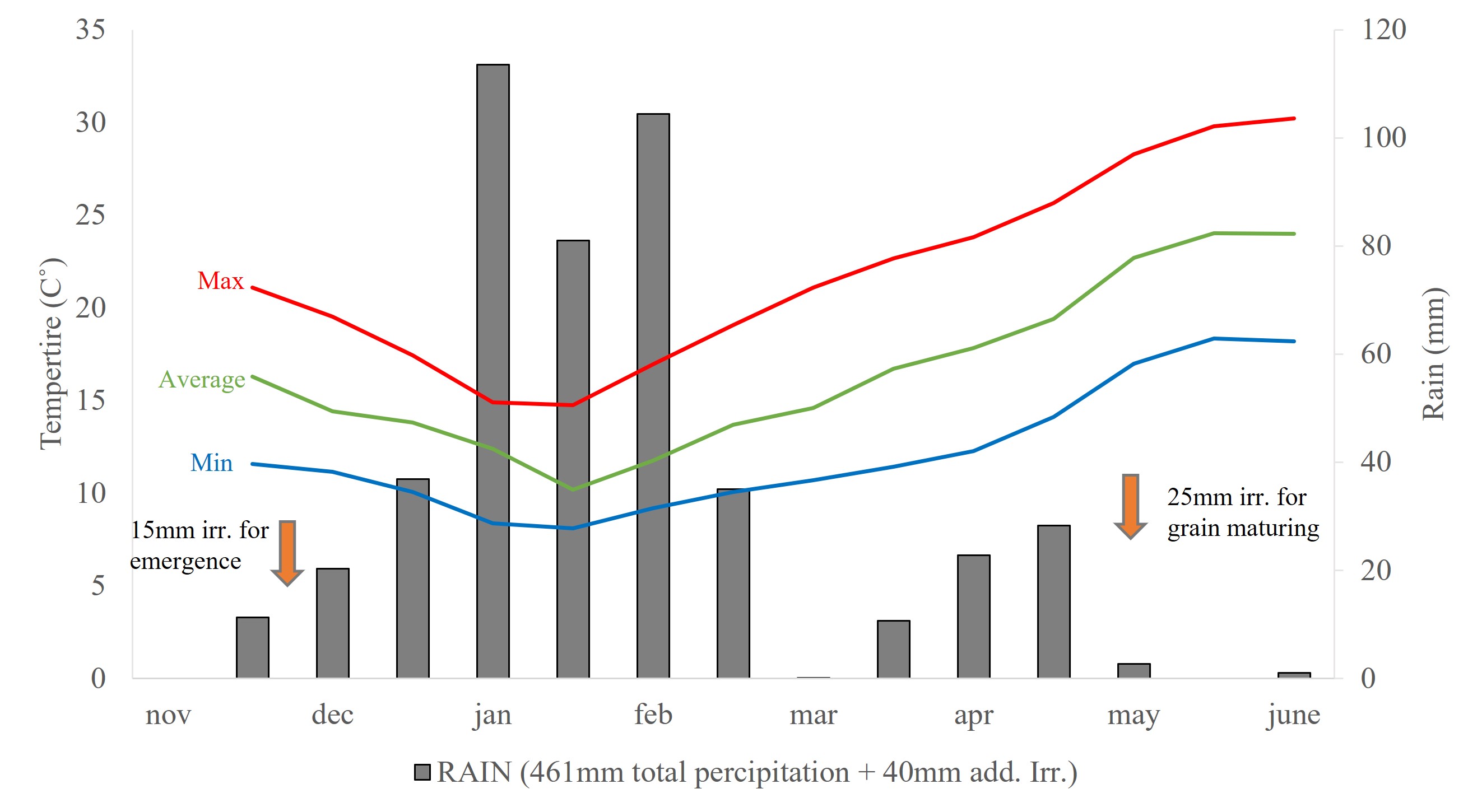

Supplement: Supplementary file 1 [file plants-11-01460-s001.zip › Supplementary Figures/Figure.S2.jpg]
